# Parent-child relationships during parenting programmes: A feasibility pilot study of the Contextualising and Learning in Mental Health Support App

**DOI:** 10.1177/13591045251354861

**Published:** 2025-07-04

**Authors:** Jasmine AL Raw, Bonamy R Oliver, Jane Gilmour, Jon Heron, Emily Midouhas

**Affiliations:** 1 Psychology and Human Development, 4919 Institute of Education, University College London, London, UK; 2 Great Ormond Street Institute of Child Health, 11700 University College London, London, UK; 3 Population Health Sciences, Bristol Medical School, 152358 University of Bristol, Bristol, UK

**Keywords:** Child behaviour, daily-life methods, feasibility, parenting, parenting programme

## Abstract

Little is known about how parent-child dynamics change when parents engage in parenting intervention programmes. To explore this, the Contextualising and Learning in Mental Health Support (CALMS) app was developed to capture daily parent-reports of these key family dynamics. This small-scale pilot study aimed to test (a) the feasibility of recruiting parents attending parenting programmes to a study of parent-child dynamics throughout a 10–12-week intervention and (b) adherence to reporting parent and child behaviours in CALMS during this period. Nine parents were recruited to complete CALMS from two parenting groups and three participated in feedback interviews. Recruitment was shown to be feasible, and adherence acceptable. Most parents reported that CALMS was easy to use, not burdensome and increased their awareness of their own and their child’s behaviours. Feasible and acceptable to parents attending parenting intervention, CALMS may have therapeutic benefits that should be explored in future research.

## Plain language summary

Little is known about how relationships between parents and children change when parents take part in parenting support programmes. To learn more, a new smartphone app called CALMS was created to allow parents to quickly record how they and their children are behaving each day. The aim of this study was to understand whether parents could be recruited from parenting programmes and how engaged parents would be with completing the CALMS app twice daily during a 10-12-week intervention. The results were positive: nearly two-thirds of parents were recruited to the study and most used the app often. Parents reported that the app was simple to use, did not take much time, and helped them notice more about their own and their child’s behaviour. In summary, the recruitment of parents to the research was achieved, daily engagement was acceptable and the app appeared to have additional benefits to parents which should be explored more in future studies.

## Introduction

Parent-child relationships are well-established mechanisms for the onset and maintenance of children’s mental-health difficulties (Patterson, 2002), and primary intervention targets (Buchanan-Pascall et al., 2018). However, studies of changes in parent-child relationships in the context of intervention are largely limited to macro-scale examinations of pre- and post-assessments (Seabra-Santos et al., 2016). Capturing ‘real time’ data during intervention using daily-life methods (e.g., ecological momentary assessment [EMA]; Schiffman et al., 2008) has the potential to improve our understanding of behavioural change by increasing data-collection efficiency, reducing recall bias and maximising ecological validity (Walls & Schafer, 2006). Daily diaries and EMA approaches—now typically delivered digitally—allow participants to report daily family life, increasing the likelihood of data being representative of real-world parent-child micro-dynamics (LoBraico et al., 2020).

To measure micro-patterns of parent and child behavioural change during intervention, parents need to be willing to complete intensive assessments during the many weeks of their programme (e.g., parenting programme), and there is a scarcity of such studies. Yet, evidence from community samples suggests day-to-day variability of parenting behaviours (e.g., warmth, praise, discipline; Fosco & Lydon-Staley, 2019; Zheng et al., 2024), suggesting that parenting may be best understood at a micro-level. Studies that explore daily transactional relationships between parents and children are especially important. Outside of the intervention context, one such study found that when parents reported more-than-usual negative parenting, their children exhibited more-than-usual externalising problems the following day (Bi et al., 2024).

Two recent studies show recruitment success for daily-diary studies with parents, and good adherence to completing once-daily reports (Bi et al., 2024; Leijten et al., 2024). However, these community (not intervention) studies were over just 14–15 days. Here, we explored feasibility of twice-daily digital diaries using the Contextualising and Learning in Mental Health Support (CALMS) app with parents attending parenting interventions over 10–13 weeks. Following participation, a subsample of parents were also interviewed for feedback, with a view to using CALMS to understand micro-change in family dynamics in larger community and intervention samples.

## Method

### Design

For this small-scale feasibility study, parents were recruited from two (fee-paying) evidence-based parenting intervention groups delivered at one site in England during April-July 2024 (Group 1: parents of children aged 4–10, 12 sessions over 13 weeks [1 week school break]; Group 2: parents of children aged 10–16, 10 sessions over 10 weeks). Clinical psychologists, parenting practitioners and parents were consulted during 2022–2024 to codevelop CALMS and the study design.

### Participants and procedure

Seventeen of 18 parents initially enrolled in the parenting groups (Group 1: *n* = 8; Group 2: *n* = 10) were eligible for recruitment, but three dropped out of the intervention before the study began, leaving 14 eligible parents. Parents completed a pre-programme questionnaire (demographics, mental health), and were then emailed download instructions for CALMS. For the duration of the intervention, participants were prompted (based on participant time-of-day preferences) to complete CALMS twice-daily. The post-programme questionnaire assessed mental health and CALMS feedback. Three parents took part in a further 30-min semi-structured interview to share their experiences and perceptions of CALMS.

### Measures

*Daily diary (CALMS):* Each log-in, parents were asked if they had been with their child since last log-in, before being prompted to select all of their child’s behaviours (e.g., tantrum, did as asked) they remembered that morning/afternoon, and all of their own parenting behaviours (e.g., shout or tell off, physical affection). They rated selected child and parenting behaviours on a visual analogue scale (1 = ‘a little’ to 100 = ‘a lot’). See Supplemental Material (SM) for a full list of CALMS questions and behaviour items.

*Semi-structured interviews* had nine questions designed to elicit parents’ experience of CALMS, focusing on utility and acceptability (Supplemental Table S2).

To measure *feasibility of recruitment*, we assessed recruitment to the study from eligible parents and attrition. *Adherence to CALMS* was captured with *full adherence* (the proportion of twice-daily completed questions out of the total number of prompts over the 10–13-week intervention: 182 prompts for Group 1; 140 prompts for Group 2) and *early adherence* (the proportion of question sets completed in the first 2 weeks, both daily and twice-daily). Early adherence was examined as a comparison with EMA studies conducted over a 2-week period. Parents reported their *experiences with CALMS* through post-intervention questionnaires and/or interview questions.

### Analyses

Basic descriptive statistics of recruitment and CALMS data investigated feasibility. Content analysis of interview transcripts investigated experiences of CALMS.

## Results

### Recruitment and adherence

**Figure 1.**
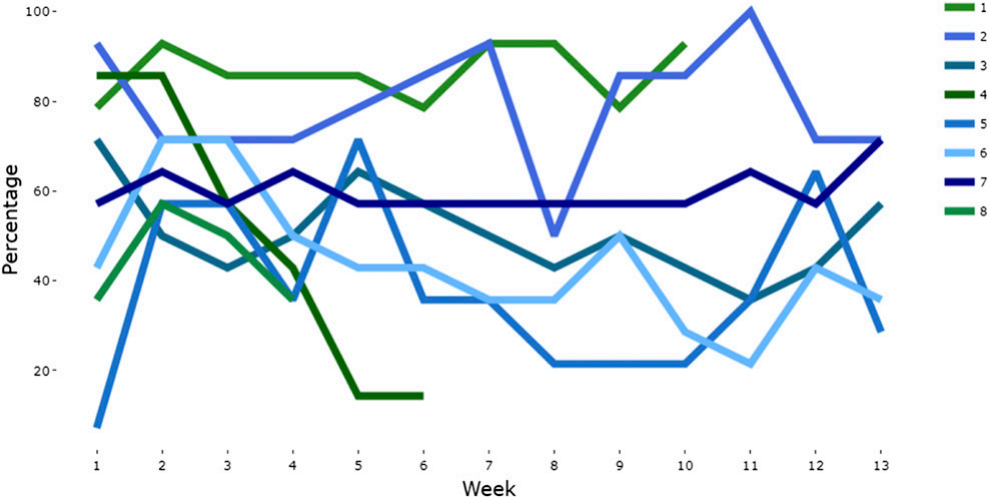
Individual trajectories of EMA full adherence percentages across 13 weeks.

The 14 eligible parents (each from a separate family) gave verbal consent to group facilitators for the researchers to contact them. Of these, nine consented to take part (64%; six from Group 1, three from Group 2) and were recruited to the study, of whom eight (89%) were retained to follow-up (Parents: 75% female, 63% White, 75% university degree, aged 19–49 years; Children: 75% male, aged 4–13 years.). Six of these eight parents engaged with CALMS for the full duration of their parenting group and are subsequently referred to as the ‘app completers’ (two parents stopped engaging around week four; see Figure 1).

All parents accessed CALMS successfully using standard written guidance. The mean percentage of question-sets completed (full CALMS adherence) was 51%, across the eight participants (ranging 18%–86% (Table 1; Figure 1]). Mean full adherence was 60% for app completers. Mean full adherence was higher on weekdays (60%) than weekend days (28%) and slightly higher for morning (46%) than afternoon (39%) question sets.

**Table 1.** Individual-level full adherence percentages for each participant.

Participant	Full adherence (%)
1	86
2	79
3	51
4*	30
5	38
6	44
7	60
8*	18

Note. Asterisks highlight participants who stopped engaging with the app after week four.

Early (first 2 weeks) adherence was 64% on average considering the number of twice-daily question sets completed out of a possible 28 (ranging 32%–86%). When considering adherence to completing at least one question set per day during the first 14 days, the mean early adherence was 79% (ranging 43%–100%).

**Figure 2.**
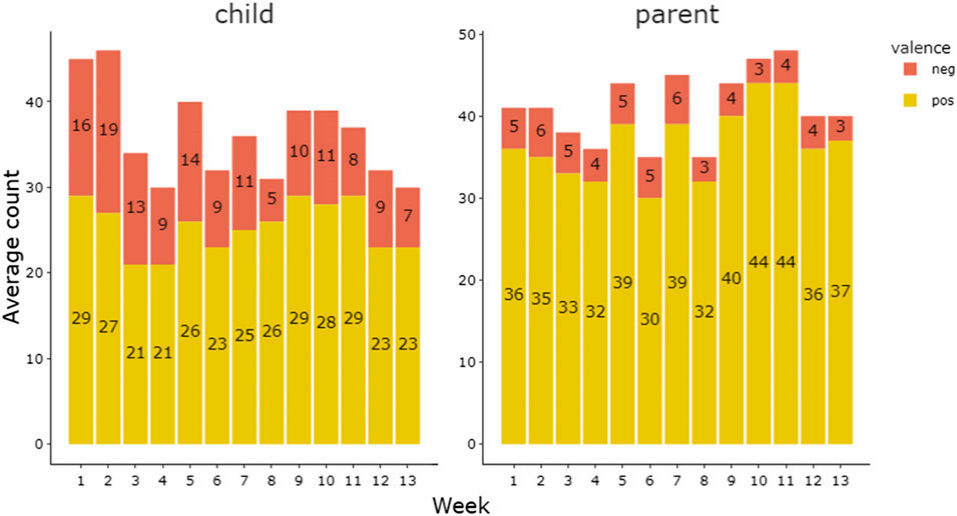
Average weekly total of positive and negative parent and child behaviours.

Parents endorsed both positive and negative child and parent behaviours, with a higher proportion of positive valence (Figure 2). All behaviours were endorsed at least once, except ‘smacking’.

### Parents’ experiences of CALMS

All three parents interviewed reported CALMS to be straightforward to navigate (e.g., “I certainly found it very, very easy to use and quite intuitive”), to cover the range of behaviours they engaged in, and to not be burdensome: (e.g., “It probably took me less than a couple of minutes…it really wasn’t difficult to fit in at all.”). Two barriers to use were routine changes at weekends (2/3 parents; e.g., “By the time I went on it, it either shut for that time period or I just completely missed it all together.”), and, for one parent, shame about reporting behaviours following negative interactions with their child.

In the post-intervention questionnaire, seven (87.5%) and six (75%) parents reported that CALMS helped them to reflect on their child’s and their own behaviours, respectively, which was reinforced in interviews (e.g., “It made me more conscious of whether I was shouting a lot”; “It made me more inclined to try and look for the positives”). Five (62.5%) parents indicated that CALMS highlighted patterns of behaviour in their relationship, also reinforced in interviews (e.g., “it definitely made me reflect on…and think about my behaviour as well as my [child’s]”). Finally, two interviewed parents reported that completing CALMS alongside the parenting course was helpful (e.g., “[CALMS] reinforced what I was learning, and it helped me to recognise my behaviours”).

## Discussion

In a small pilot study, we assessed the feasibility of CALMS, a smartphone-app designed to record parents’ twice-daily reports of family dynamics during parenting intervention. Almost two-thirds of eligible participants were recruited and engaged with CALMS throughout intervention. Questionnaires and interviews suggested parents found CALMS acceptable, easy to use, not burdensome alongside their intervention programme, and questions aligned well with behaviours they recognised in themselves and their child.

Around half of the twice-daily questions were completed throughout intervention. Other daily-diary studies have reported around 80% adherence (e.g., Aunola et al., 2013; Bi et al., 2024). However, many community sample studies use shorter periods of data collection (e.g., 2 weeks) with only once daily-diary entries. Early adherence—the proportion of participants engaging at least once-per day in the first 2 weeks—was more closely aligned with the recommended 80%, suggesting reducing expected entries to once-per-day may increase adherence. Additionally, to raise adherence, we will consider increasing the number of reminders and reducing the number of expected entries over the intervention period. As weekend adherence was lower than weekdays due to family routine changes, dropping weekend prompts may increase engagement. However, this could introduce confounds and systematic missingness which we will need to explore. Additionally, intrinsic incentives (e.g., data visualisation) have been shown to increase adherence (Hsieh et al., 2008). Consideration of these strategies for CALMS are under way, but require careful co-development with practitioners, clinicians and parents, due to the sensitive nature of the data.

In this small sample, CALMS captured a range of both positive and negative behaviours from parents in line with our expectations of parent-child interactions. All behaviours except ‘smacking’ were endorsed, perhaps indicating under-reporting (Department of Epidemiology and Public Health, University College London, 2024), and, given one parent reported shame related to reporting negative parenting biases in our data due to social desirability (Grimm, 2010). Social desirability is a common concern in the parenting literature, particularly negative rather than positive behaviours, and ways of reducing socially desirable responses are crucial for the entire field. The positive feedback from parents on CALMS leads us to believe that it is not worse, and may even be better, than other methods.

CALMS was implemented as a data-collection tool, yet interviews suggested potential therapeutic effects due to the self-reflection encouraged by completing it. Indeed, tracking daily behaviours in CALMS seemed to increase self-awareness which might lead to positive behavioural changes, something evidenced in the healthcare literature (Hennesey et al., 2020; König et al., 2022). Whether these effects extend to parenting behaviours is somewhat unclear (Fuller-Tyszkiewicz et al., 2020). Nevertheless, CALMS may have potential beyond its intended use, and future work exploring this possibility in community and intervention samples is of interest.

A strength of our study is that we tested a co-produced novel data collection tool to measure daily parent-child dynamics during intervention. CALMS can also be used in a community sample of parents given the applicability of behaviours to everyday family life. Additionally, our study involved the sustained collection of daily data from parents throughout a nearly three-month intervention compared to most EMA studies taking place over only 2 weeks. Our study also has limitations. Parenting groups were paid for, such that parents were unlikely to be socio-economically representative of those in other parenting-intervention settings. To increase diversity of recruitment, we are currently developing a wider network of such settings. Relatedly, having only three interview participants limited the range of views captured, and, since we were unable to interview parents who chose not to participate, our understanding of barriers to engagement was restricted.

Overall, participants’ perceptions of CALMS were positive, findings supported feasibility and acceptability for parents taking part in parenting intervention. Findings suggest that CALMS may augment the positive effects of parenting intervention, demonstrating its future potential as an additional form of support easily integrated into existing practice.

## Supplemental Material

Supplemental material - Parent-child relationships during parenting programmes: A feasibility pilot study of the Contextualising and Learning in Mental Health Support app Supplemental material for Parent-child relationships during parenting programmes: A feasibility pilot study of the Contextualising and Learning in Mental Health Support app by Jasmine A. L. Raw, Bonamy R. Oliver, Jane Gilmour, Jon Heron and Emily Midouhas in Clinical Child Psychology and Psychiatry

## Data Availability

Data from this study are for pilot research and will be made available along with additional data after the main study has been completed.*
