# Supplementary material for: Parent-child relationships during parenting programmes: A feasibility pilot study of the Contextualising and Learning in Mental Health Support App
Source: Clin Child Psychol Psychiatry. 2025 Jul 4;30(4):865–73. doi: 10.1177/13591045251354861 (PMC12436981; doi:10.1177/13591045251354861)
Supplement: Supplemental material - Parent-child relationships during parenting programmes: A feasibility pilot study of the Contextualising and Learning in Mental Health Support app [file sj-pdf-1-ccp-10.1177_13591045251354861.pdf]

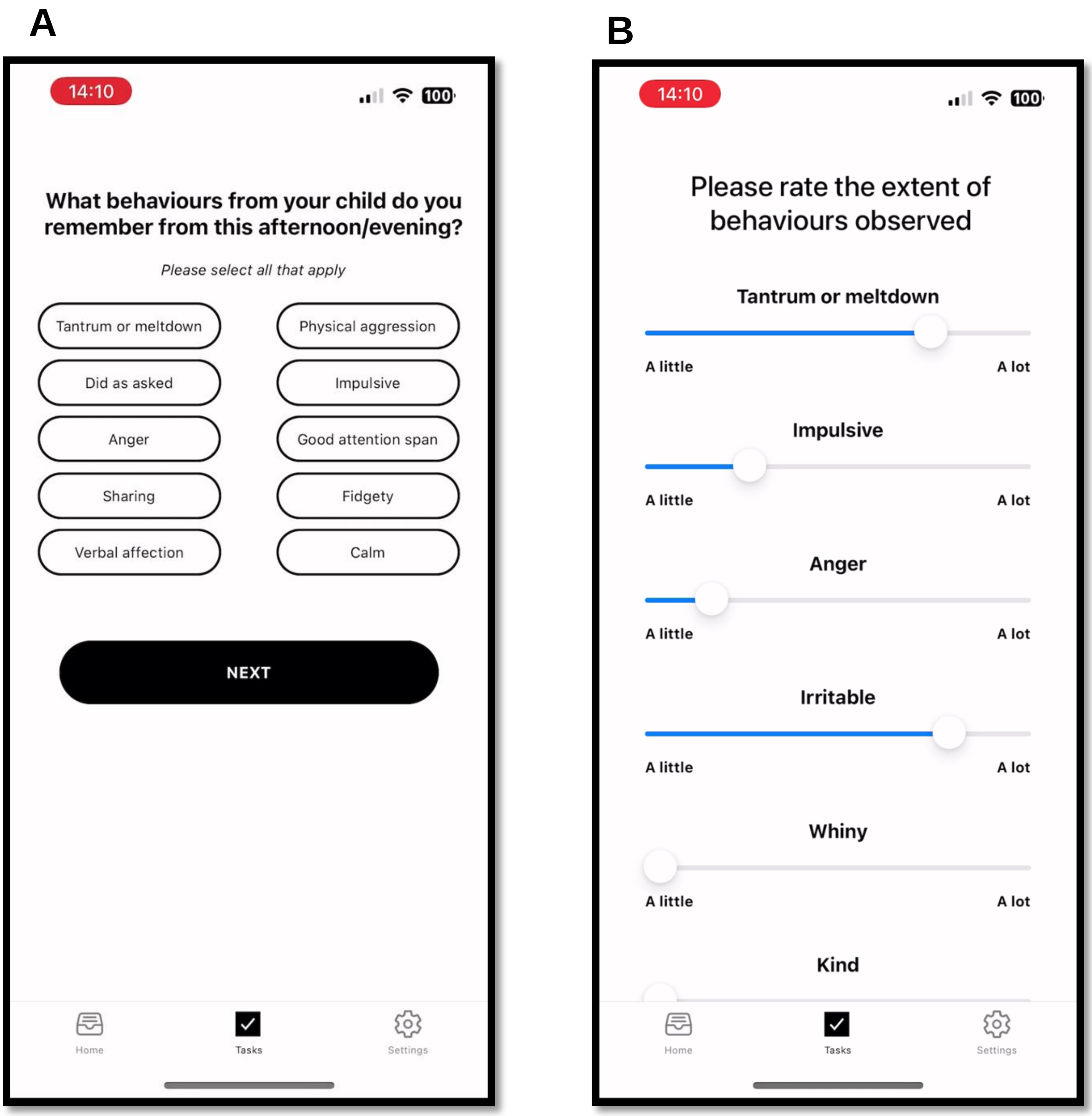

Figure S1. A) An example screen of some of the child behaviour options that parents could select. B) An example screen containing the visual analogue scale for each behaviour selected.

Table S1

Table of questions asked via the CALMS smartphone application

Question	Response/Scale	AM/PM	Day
Have you been with your child since the last survey?	Yes/no	AM/PM	Daily
Rate your child's sleep quality last night.	1 "*not at all well*" - 10 "*very well*"	AM	Daily
Rate your stress level since the last survey.	*1 "not at all stressed" - 10 "very stressed"*	AM/PM	Daily
Rate your mood since the last survey.	*1 "very low mood" - to 10 "very good mood"*	AM/PM	Daily
What behaviours from your child do you remember from this morning? *(Tick all that apply).*	- Tantrum or meltdow n<br>- Physical aggression<br>- Follow instructions<br>- Impulsive<br>- Anger<br>- Good attention span<br>- Sharing<br>- Fidgety<br>- Verbal affection<br>- Calm<br>- Irritable<br>- Physical affection<br>- Argue or chat-back<br>- Noisy<br>- Considerate<br>- Easily distracted<br>- Helpful<br>- Whine<br>- Kind<br>- Not follow instructions	AM/PM	Daily
Please rate the extent of behaviours observed.	1 "A little" - 100 "A lot"	AM/PM	Daily
What behaviours of yours do you most remember from this morning? *(Tick all that apply).*	- Organised<br>- Praise<br>- Smack<br>- Encourage<br>- Shout<br>- Physical affection<br>- Verbal affection<br>- Make the time fun<br>- Irritable<br>- Give limited choices<br>- Tell off<br>- Give positive attention<br>- Nag<br>- Criticize<br>- Set limits<br>- Help child to be calm<br>- Have time with child		
Please rate the extent of your behaviours.	1 "a little" - 100 "a lot"	AM/PM	Daily
I know why my child acts the way he or she does.	1 "*strongly disagree*" - 100 "*strongly agree*"	PM	Friday
How confident are you in	1 "*not very confident*" - 100 "*very confident*"		

your parenting this week?

Table S2

Table of questions included in the semi-structured interviews

**Questions**
1. We would really like to know if you thought the CALMS smartphone application was user-friendly. For example, was it easy to use and was it easy to navigate around?
2. Did you find any questions difficult to understand or answer?
3. Did you experience any technical difficulties while using the app?
4. We would like to understand more about the barriers that may have prevented you from engaging with the app. Therefore, we want to know if there was anything that prevented you from engaging with the app? For example, you were too busy, it took too long to answer the questions, or you became fatigued with answering them?
5. Overall, is there anything else that you think we could improve on? This can include the questions we ask or even the layout or appearance of the application.
6. Did the app have any impact on your relationship with your child?
7. Did it make you more aware of your child's behaviours?
8. Do you think it influenced the way you parented at all?
9. Did you notice changes in your child's behaviours and how much do you think this was down to the app?

Table S3

Table of scales asked in the pre- and post-parenting group Qualtrics surveys

Scale/ Question	Response/ Scale	Reference
Strengths and Difficulties Questionnaire	0 = Not True<br>1 = Somewhat True<br>2 = Certainly True	Goodman, R. (1997). *Strengths and Difficulties Questionnaire (SDQ)* [Database record]. APA PsycTests.
Confusion, Hubbub and Order Scale (CHAOS)	5-point Likert scale:<br>- None at all<br>- A little<br>- A moderate amount<br>- A lot<br>A great deal	Matheny, A. P., Wachs, T. D., Ludwig, J. L., & Phillips, K. (1995). Bringing order out of chaos: Psychometric characteristics of the confusion, hubbub, and order scale. *Journal of Applied Developmental Psychology, 16*(3), 429–444. https://doi.org/10.1016/0193-3973(95)90028-4
Kessler K6	5-point Likert scale<br>- All of the time<br>- Most of the time<br>- Some of the time<br>- A little of the time<br>None of the time	Kessler, R.C., Andrews, G., Colpe, L.J., Hiripi, E., Mroczek, D.K., Normand, S.L., Walters, E.E., Zaslavsky, A.M. (2002) Short screening scales to monitor population prevalences and trends in non-specific psychological distress. Psychological Medicine, 32, 959–976, DOI: 10.1017/S0033291702006074

CALMS app related questions

1. To what extent, if at all, did the use of the **CALMS smartphone app** make you more aware of how you interact with your child?	- Not at all<br>- Somewhat<br>- A lot

2. Can you expand on why or why not? *Please tick all that apply.*	- I often experienced technical difficulties with the app<br>- I struggled to find the time to answer the app<br>- It made me reflect on my feelings and behaviours including the positive things I had done<br>- I was already aware of how I interact with my child – the app did not help to make me more aware<br>- It helped me think about the behaviours I could improve on<br>- Other. Please briefly explain in a few sentences or less.
3. To what extent, if at all, did the use of CALMS make you more aware of your child's behaviours?	- Not at all<br>- Somewhat<br>- A lot
4. Can you expand on why or why not? *Please tick all that apply.*	- It helped me to identify my child's feelings and behaviours better including the positive things they had done<br>- It was helpful to record my child's behaviour each day to understand what they struggle with the most<br>- I was already aware of my child's behaviour – the app did not make me more aware<br>- Other. Please briefly explain in a few sentences or less.
5. To what extent, if at all, did the use of CALMS change your parenting behaviour?	- Not at all<br>- Somewhat<br>- A lot
6. Can you expand on why or why not? *Please tick all that apply.*	- I feel like my parenting behaviours have stayed the same<br>- It helped me to consider some positive ways of interacting with my child such as praising or helping them, giving them positive attention, hugging or kissing them, playing with them and having some quality time with them<br>- It helped me to identify areas of my parenting that I could improve on such as not criticising or shouting at them<br>- Other. Please briefly explain in a few sentences or less.

7. To what extent, if at all, did the use of CALMS change your child's behaviour?
   - Not at all
   - Somewhat
   - A lot

8. Can you expand on why or why not? *Please tick all that apply.*
   - I feel like my child's behaviour has stayed the same
   - Other. Please briefly explain in a few sentences or less

9. To what extent, if at all, did the use of CALMS change your interactions with your child?
   - Not at all
   - Somewhat
   - A lot

10. Can you expand on why or why not? *Please tick all that apply.*
    - My interactions with my child have stayed the same
    - It made me reflect on how I could have done things differently when my child was struggling with their behaviour
    - It highlighted patterns of behaviour between me and my child that we can work on together
    - It has brought us closer together
    - Other. Please briefly explain in a few sentences or less

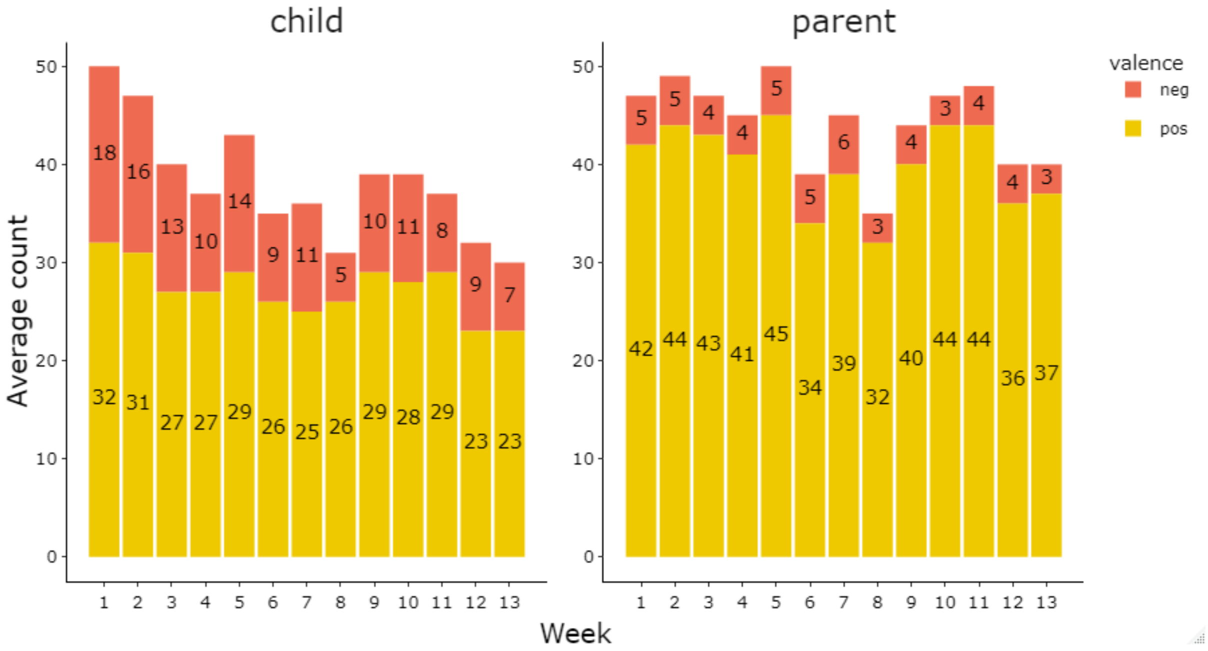

Figure S2. *Average weekly total of positive and negative child behaviours from the six participants who continued their engagement with the app throughout their parenting course.*
